# Disease burden among patients with Arginase 1 deficiency and their caregivers: A multinational, cross‐sectional survey

**DOI:** 10.1002/jmd2.12456

**Published:** 2024-10-29

**Authors:** Sara Olofsson, Sofia Löfvendahl, Julia Widén, Lena Jacobson, Peter Lindgren, Karolina M. Stepien, Jean‐Baptiste Arnoux, Maria Luz Couce Pico, Elisa Leão Teles, Mattias Rudebeck

**Affiliations:** ^1^ The Swedish Institute for Health Economics, IHE Lund Sweden; ^2^ Immedica Pharma AB Stockholm Sweden; ^3^ Karolinska Institutet Stockholm Sweden; ^4^ Salford Royal Organization, Northern Care Alliance NHS Foundation Trust Salford UK; ^5^ Necker Hospital Paris France; ^6^ Hospital Clinico Universitario de Santiago de Compostela, IDIS, MetabERN Santiago de Compostela Spain; ^7^ Centro Hospitalar Universitário de São João, MetabERN Porto Portugal

**Keywords:** ARG1‐D, arginase 1 deficiency, caregiver, disease burden, patient perspective, urea cycle disorder

## Abstract

Arginase 1 deficiency (ARG1‐D) is an ultrarare, metabolic disease which may cause spastic paraplegia, cognitive deficiency, seizures, and ultimately severe disability. The aim of this study was to assess disease burden in ARG1‐D by performing a cross‐sectional survey of patients with ARG1‐D and their caregivers in four European countries (France, Portugal, Spain, and the United Kingdom). Patients were enrolled at participating clinics and data were collected using a web‐based questionnaire. The findings indicate that there is a significant share of patients who experience severe cognitive and mobility impairment but also that there is a considerable variance in symptom severity among patients. Disease management was mostly in line with treatment guidelines and self‐reported adherence to treatment was reported to be high among a majority although following diet restrictions was perceived as difficult. However, despite this, since a large share of patients experienced severe cognitive and mobility impairment an unmet need among this patient population is suggested. The introduction of disease‐modifying therapies and early identification and diagnosis may help alleviate the disease burden associated with ARG1‐D in the future.

**Synopsis**

Despite disease management in line with treatment guidelines, a large share of patients with arginase 1 deficiency experience severe cognitive and mobility impairment, suggesting an unmet need among this patient population.

## 1 INTRODUCTION

Arginase 1 deficiency (ARG1‐D; OMIM # 207800), also known as hyperargininaemia, is an ultrarare progressive, autosomal recessive, metabolic disease, affecting approximately 1 in 726 000 to 950 000 births.^1^, ^2^ ARG1‐D is characterized by a deficiency of the final enzyme of the urea cycle, arginase 1 (EC3.5.3.1), leading to elevated plasma arginine (pArg) levels and episodic hyperammonemia. Manifestation of the disease includes spastic paraplegia, cognitive deficiency, epilepsy and ultimately severe disability.^3^ In contrast to other urea cycle disorders, birth and early infancy are relatively normal and the first symptoms are generally noted between 2 and 4 years of age.^4^, ^5^ In settings without newborn screening (NBS) for ARG1‐D or where sibling screening in families with previously affected children has not been performed, the diagnosis is often delayed and triggered by the onset of early childhood symptoms. Also, misdiagnosis is common, where common initial incorrect diagnoses have been reported to be hereditary spastic paraplegia or cerebral palsy (CP) due to the resemblance in symptoms.^6^, ^7^, ^8^

The current standard treatment for ARG1‐D includes severe dietary protein restriction (which may require essential amino acid (EAA) supplementation) with the aim to reduce pArg levels together with nitrogen scavengers when needed and symptomatic treatments for the disease manifestations.^8^, ^9^ Recent studies describing the nature and management of ARG1‐D include a review of published case reports (*n* = 157),^6^ a retrospective review of medical records (*n* = 6),^10^ and a review of the background characteristics of a clinical trial (*n* = 32).^11^ These studies show that, despite standard treatment, most patients continue to have elevated pArg levels and continue to experience progression of clinical manifestations. The studies also confirm that diagnosis is often delayed and show that treatment adherence may be problematic due to severe diet restrictions. Although this may contribute to the inadequate effect of standard treatment, the authors of these studies conclude that there is an unmet need for disease‐modifying therapies as current treatment options target dietary arginine intake but cannot affect endogenous arginine production.^6^, ^10^ Recently, pegzilarginase was approved by the European Medicines Agency (EMA) as the first disease‐modifying treatment, an arginine lowering enzyme therapy.^12^

There is a lack of studies reporting the disease burden of ARG1‐D from the perspective of patients and their caregivers. The objective of this study was to assess disease burden in ARG1‐D by performing a descriptive cross‐sectional survey of patients with ARG1‐D and their caregivers in four European countries. The study is, to our knowledge, the first survey performed in ARG1‐D and will add to the existing literature by providing more insights on self‐reported variables (e.g., treatment adherence, symptom severity and caregiver burden) that may have an impact on disease management and treatment outcomes. Moreover, the study was designed to categorize disease severity among patients by both mobility and cognitive function. Finally, the study will provide an updated picture of the natural history of the disease with the currently available treatment options.

## 2 METHODS

This study was designed as a descriptive cross‐sectional, international, multi‐centre survey conducted in France, Portugal, Spain and the United Kingdom (UK).

### 2.1 Study sample

Patients with an ARG1‐D diagnosis (and their caregivers) connected to the participating or referring clinics were eligible for inclusion. Patients treated with pegzilarginase as part of an early access program were eligible given that treatment period was relatively short as the program was recently introduced, whereas patients participating in the longer‐term treatment interventional clinical trials (including extension studies),^8^, ^13^ were excluded from the study, as were patients/caregivers that could not read and write in the country‐specific language. However, in case a patient was unable to read and write, a caregiver could respond on their behalf.

### 2.2 Survey instrument

Data were collected using a web‐based questionnaire developed in collaboration with the clinical expert team conducting this study, including metabolic specialists and paediatricians. The questionnaire consisted of 12 sections with the first part (Part I) concerning the patient and the second part (Part II) concerning the caregiver. Part I included questions on patient demographics, disease and symptoms, health care resource use, diet and medication, professional caregiving and assistance, adult patients' (16 years+) ability to work and health‐related quality‐of‐life (HRQoL). Part II included questions on caregiver demographics, amount of caregiving by the family, patients' symptoms, caregivers' ability to work and caregiver's HRQoL and caregiver burden. The questionnaires are available in supplemental information (S1, questionnaire for caregivers; S2, questionnaire for patients).

Resource use and ability to work related to ARG1‐D were assessed for the last 12 months. Details on resource use, ability to work and HRQoL are reported in a separate article covering the cost and utility loss associated with ARG1‐D.^14^

Patients' symptoms were assessed by the caregiver using the Gross Motor Function Classification System (GMFCS) for motor function and adapted scales for cognitive function. Questions around the patient's symptoms were included in the caregiver Part II due to the sensitive or challenging nature of these questions, making it inappropriate to directly posing specific symptom‐related questions to the patient themselves.

The GMFCS is a five‐level classification, where higher score indicate worse gross motor function, developed for use in CP, a population presenting with similar neurologic features to ARG1‐D. GMFCS has also been used in studies with ARG1‐D.^8^, ^15^ The tool differentiates children based on the child's current gross motor abilities, limitations in gross motor function, and need for assistive technology and wheeled mobility. The instrument is adapted to the child's age and available in versions for children who are 2–4 years, 4–6 years, 6–12 years and 12–18 years.^16^ The GMFCS is not used for children below the age of 2 years. The GMFCS for children 12–18 years was used to measure motor function in adults.

Cognitive function was measured by a study specific scale developed in collaboration with medical expertise and adapting selected dimensions from validated instruments (e.g. The Denver Development Screening Test). The scale included 13 questions on the patients' ability to communicate, socialize, learn/think and solve problems and perform activities of daily living (see supplement for more details). The ability was rated on a scale from 0 (no problems) to 4 (cannot do at all). Caregivers were asked to rate the ability of the patient in comparison to other children/adults of the same age. The respondent had the option of responding “Not relevant” (e.g., ability to read and write for young children).

The Zarit Burden Interview (ZBI) Short (12 items) was used to measure caregiver burden. The instrument includes 12 questions related to caregiving (e.g., “Do you feel that because of the time you spend with your relative you don't have enough time for yourself”) that is rated on a scale from 0 (never) to 4 (nearly always).

### 2.3 Data collection

When a patient fulfilling the eligibility criteria arrived at the clinic for their appointment, the patient or the caregiver was informed about the study and given an invitation letter. Alternatively, the patient or caregiver was informed over telephone by the health care staff and the invitation letter was sent to them by e‐mail approximately 2 weeks before the clinic visit. The patient and/or caregiver completed the questionnaire at the clinic, or at home, by using the link included in the invitation letter. The survey was translated to local languages by local consultants in France, Portugal and Spain and programmed to a web‐based version by Enkätfabriken (Göteborg, Sweden) using the platform LimeSurvey©.

**FIGURE 1:**
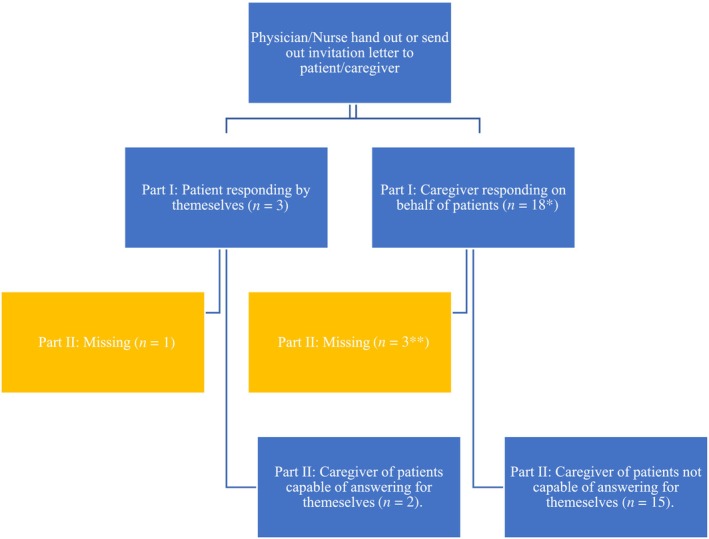
Survey respondents and missing data. *17 caregivers responding for 18 patients (1 caregiver responded for 2 patients). **In addition, there were 3 caregivers responding that they had other child(ren) with ARG1‐D but who did not respond to Part I for these children.

A total of 43 patients (France: 9, Portugal: 10, Spain: 7 and UK: 17) and their caregivers were invited to participate in the study. In total, there were 21 responses for Part I and 16 responses to Part II. Figure 1 summarizes the respondents and missing data. Caregivers responded to Part I (*n* = 17) for children (i.e., below 16 or 18 years, according to local regulation) and adults not capable of answering for themselves before responding to Part II (*n* = 14, 3 responses missing). Adult patients capable of answering Part I (*n* = 3), invited their caregivers to respond to Part II (*n* = 2, one response missing). Caregivers with more than one child with ARG1‐D (*n* = 4) were first asked to complete Part I for their youngest child. Thereafter, they completed Part I for their older child(ren) which occurred automatically in the web‐based platform (*n* = 1, 3 missing).

Data were collected between June 16 and August 13, 2023.

### 2.4 Data analysis

The data were summarized using standard descriptive measures as appropriate; proportions for categorical variables and mean and standard deviation for continuous variables. Data were presented for all countries merged.

GMFCS scores were missing for four patients as their caregiver did not complete Part II, which included the relevant questions. A GMFCS score was instead assessed based on the responses to other relevant questions in Part I (wheelchair use, mobility dimension in the EQ‐5D) for three patients. One additional patient was not eligible for GMFCS scoring due to age (<2 years old). To avoid presenting data for individual observations, costs for GMFCS levels 3, 4 and 5 were combined and presented as one.

A total cognitive score was calculated by summarizing the score (0–4) of each separate question (in total 13 questions). The responses ‘Don't know’ and ‘Not relevant’ were coded as 0. Cognitive score ranges from 0 (0 for all questions) to 52 (4 for all questions).

A caregiver burden score ranging from 0 to 48, where 0 indicates least caregiver burden and 48 most caregiver burden, was calculated based on the ZBI instrument using suggested guidelines for scoring.^17^ Scores were classified into three groups according to recommendations^18^: none to mild burden (score 0–9), mild to moderate burden (score 10–20) and high burden (score > 20).

## 3 RESULTS

### 3.1 Sample characteristics

A majority (76%) of 21 patients were females, the mean age was 16.7 years (min: 0; max: 49) and 12 (57%) were below 16 years of age (most common definition of child) (Table 1). Almost all (86%) lived with their parents. Specialized education was received or had been received by 9 patients (43%) and none of the adult patients were used. Six patients (29%) had been diagnosed by NBS (mean age 7.7 years, min: 0; max: 14). The mean age at diagnosis was 3.8 years (min: 0; max: 16, *n* = 16) in the entire sample and 6.0 years (min: 0; max: 16, *n* = 10) among patients not diagnosed by NBS. Three patients (14%) were misdiagnosed with a different condition before being diagnosed with ARG1‐D (1, 2.5 and 13 years diagnosis delay). These diagnoses were spasticity/paraplegia, cerebral paresis and squint (Table 2).

**TABLE 1:** Sample characteristics.

Variable		Number of patients
Mean age (*N* = 21)	16.7 (11.7) years	21
Median (min, max)	14.0 (0, 49) years	21
Female (*N* = 21)	76%	16
Current living arrangement (*N* = 21)		
Living with parent(s)	86%	18
Living in own home	10%	2
Other	5%	1
Current main occupation (*N* = 21)		
Below school age	5%	1
Primary or secondary school	47%	10
University	5%	1
Employed or self‐employed	0%	0
Sick leave or early retirement	0%	0
Unemployed or looking for work	5%	1
Other^a^	38%	8
Specialized education (*N* = 21)		
Yes^b^	43%	9
No	48%	10
Do not know	10%	2

Abbreviations: Q1, quartile 1; Q3, quartile 3; SD, standard deviation.

^a^ Instituts médico‐éducatifs (IME),”Educación Infantil” and out of school. Unable to work, day centre, établissement polyhandicap, centre spécialisé.

^b^ Exemples were Instituts médico‐éducatifs (IME), Special Need Education (SEN), Unités localisées pour l'inclusion scolaire (ULIS). There were five patients with age range 8–18 currently in special education, i.e., currently receiving this education.

**TABLE 2:** Disease characteristics.

Variable		Number of patients
Age at diagnosis (*N* = 16)		
Mean (SD)	3.8 (4.8) years	16
Median (Q1, Q3)	2.3 (0, 6.5)	
Min; max	0; 16	
Age at first sign of cognitive deficiency (*N* = 13)		
Mean (SD)	5.0 (3.5) years	13
Median (Q1, Q3)	4 (3, 6)	
Min; max	2; 15	
Mean (SD) age at first sign of limited mobility (*N* = 13)		
Mean (SD)	9.2 (12.6) years	13
Median (Q1, Q3)	5 (3, 10)	
Min; max	0; 48	
Mobility and cognitive ability (*N* = 21)		
Limited mobility only	10%	2
Cognitive dysfunction only	10%	2
Both	52%	11
None	24%	5
Do not know	5%	1
Spasticity (*N* = 21)		
Lower limbs	57%	12
Upper limbs	0%	0
Both lower and upper limbs	5%	1
No	38%	8
Experienced seizures (*N* = 21)		
Yes	29%	6
No	62%	13
Do not know	10%	2
GMFCS (*n* = 19)		
1 (best level)	42%	8
2	26%	5
3	5%	1
4	21%	4
5 (worst level)	5%	1
Cognitive score (*n* = 17)		
0–9 (best level)	41%	7
10–19	18%	3
20–29	12%	2
30–39	12%	2
40–52 (worst level)	18%	3
Mean score (SD)	18.9 (18.0)	17

Limited mobility and/or cognitive deficiency were present in 15 patients (71%), spasticity was present in 13 patients (61%) and 6 patients (29%) had a history of seizures. Movement ability according to GMFCS (data available for 19 patients) was at the mildest/best level (1) for 8 patients (42%), at a moderate level (2) for 5 patients (26%) and at a more severe level (3–5) for 6 patients (32%). Among the six patients diagnosed with NBS, five (83%) reported being at level 1 (best level) on the GMFCS and one was under the age eligible for the GMFCS (2 years of age). The mean cognitive score for the entire sample was 18.8 (on a scale from 0 to 52, where 0 = best), 11.2 for patients diagnosed by NBS and 28.2 for patients not diagnosed by NBS.

### 3.2 Disease development

**FIGURE 2:**
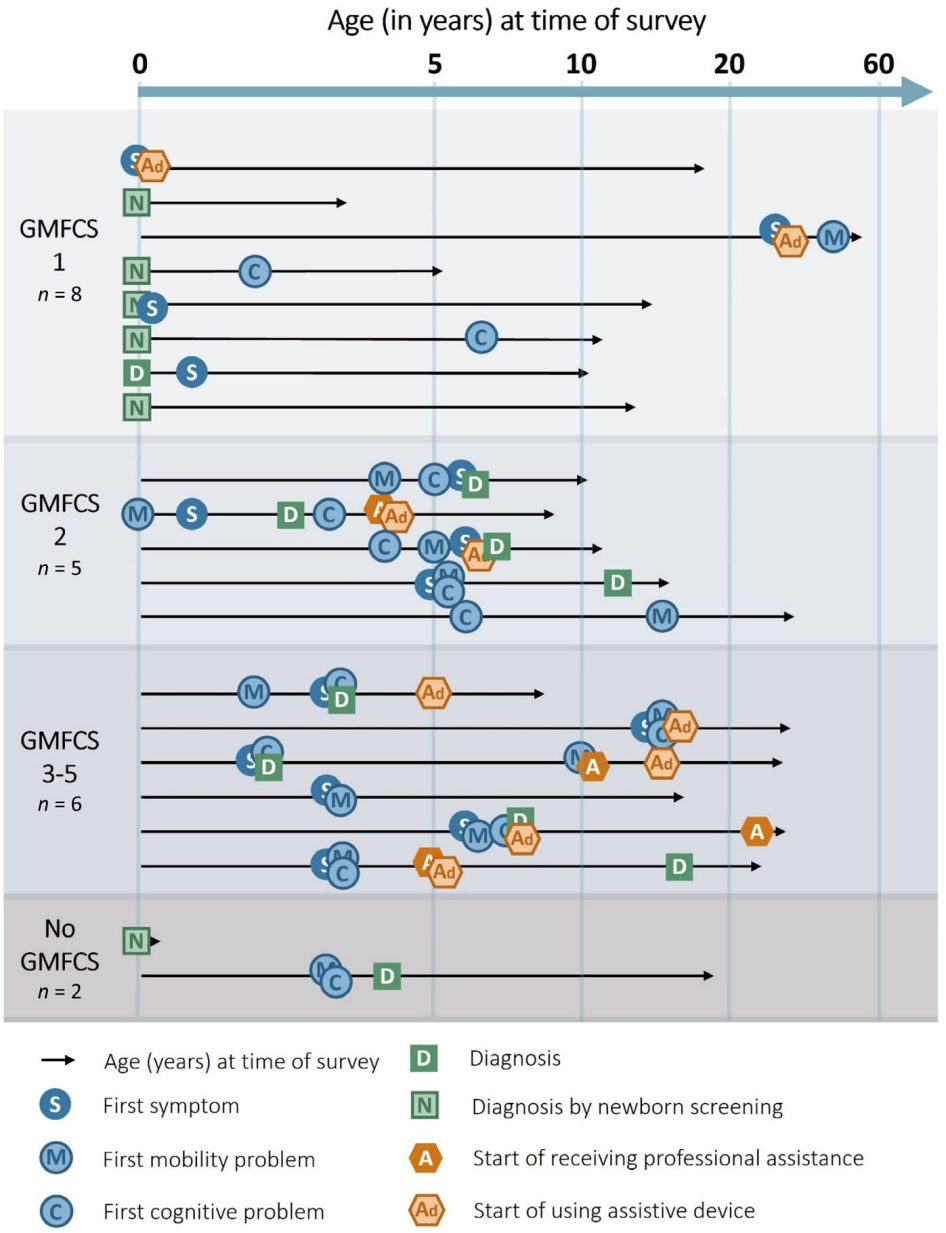
Disease development. No marker does not necessarily imply absence of symptoms or assistance. These respondents may have reported symptoms or assistance but not the age at which these started.

The first symptom appeared in childhood for most patients, before or around 5 years of age (Figure 2). However, there were a few exceptions with first symptoms in adolescence or early adulthood. The type of first symptoms varied, with some patients experiencing mobility limitations first and others cognitive deficiency. Among patients with GMFCS 1 (*n* = 8), caregivers for four patients (50%) reported no cognitive problems. All patients with a GMFCS levels 4–5 had a relatively high (worse) cognitive score. Except for the patients diagnosed by NBS (where first symptoms appear *after* diagnosis), there were in general a delay of approximately 2.5 years from the first symptom to diagnosis. Use of walking aids or devices was reported for nine patients (43%), whereof age at initial use was as early as 5 years old for one patient, 8 years for six patients and 12 years for two additional patients.

Personal assistance was provided to four patients reporting both mobility and cognitive symptoms.

### 3.3 Disease management

During the last 12 months, six patients (29%) visited the emergency department due to their ARG1‐D diagnosis (Table 3). An equally large share, with five of the six patients among those who visited the emergency department, had been hospitalized for an average of 8 days. A majority (87%) had made health care visits due to ARG1‐D during the last 12 months. The most frequently visited health care staff included metabolic specialist (70%, 4.2 visits), dietician (52%, 5.3 visits), neurologist (43%, 1.4 visits) and physiotherapist (38%, 26.3 visits). Almost all patients (90%) had done a blood sample test during the last 12 months, and around a third (33%) had done an ultrasound. A smaller share (19%) had done ECG, EEG (10%), MRI scan (10%), or CT scan (5%). Eleven (52%) patients received treatment with botulinum toxin, where 45% reported presence of spasticity.

**TABLE 3:** Disease management due to ARG1‐D during the last 12 months.

Variable	Proportion (%) of patients	Number of patients	Mean (SD) number of visits among those with any visits
Emergency department at the hospital, (*N* = 21)			
Yes	29%	6	1.8 (0.75)
No	71%	15	
Hospitalization (*N* = 21)			
Yes	29%	6	
No	71%	15	
No. of hospitalization (N = 6)			
1 stay	67%	4	
2 stays	17%	1	
3 stays	17%	1	
Hospitals days, mean (SD)	–	6	7.7 (7.7) days
Health care staff (*N* = 21)			
Visits to any health care staff	87%	18	Not applicable
Neurologist	33%	7	1.4 (0.8)
Pediatrician	10%	2	2.3 (2.1)
Endocrinologist	62%	13	4.2 (3.5)
Geneticist	0%	0	0
General practitioner/pediatrician	5%	1	3.0 (−)
Nurse	21%	4	22.8 (34.3)
Physiotherapist/Rehabilitation specialist	29%	6	26.3 (31.0)
Occupational therapist	10%	2	20.0 (21.2)
Psychologist	24%	5	13.4 (20.7)
Dietitian	48%	10	5.3 (4.2)
Speech therapist	10%	2	18.0 (24.0)
Other	14%	3	17.7 (16.2)
Tests (*N* = 21)			
Blood sample	90%	19	4.9 (7.8)
Ultrasound	33%	7	1.9 (1.5)
Electrocardiogram (ECG)	19%	4	1.8 (1.0)
Electroencephalogram (EEG)	10%	2	1.0 (0)
MRI scan	10%	3	2.7 (2.9)
CT scan	5%	1	3.0 (−)
Other	24%	5	4.8 (5.0)
Do not know	10%	2	
Injection treatments with botulinum toxin (*N* = 21)	52%	11	10.1 (28.8) treatment times
Surgical treatment for muscle stiffness (*N* = 21)	0%	0	0
Special diet prescription (*N* = 21)			
Yes	95%	20	
No	5%	1	
Regularly consumption of essential amino acid (EAA) supplementation (*N* = 21)			
Yes	71%	15	
No	24%	5	
Do not know	5%	1	
Manage to follow diet guidelines last 7 days (*N* = 20)			
Yes, all meals	65%	13	
Most of the time	15%	3	
Sometimes	10%	2	
Do not know	10%	2	
*Missing*		*1*	
Easy/difficult to follow diet (*N* = 20)			
Very easy	10%	2	
Easy	35%	7	
Difficult	35%	7	
Very difficult	10%	2	
Don't know	10%	2	
*Missing*		*1*	
Problems swallowing and/or self‐feed (*N* = 21)			
No problems	67%	14	
Problems swallowing	5%	1	
Problems self‐feed	24%	5	
Don't know	10%	2	
Pharmaceuticals used during the last 12 months (*N* = 21)			
Any use of sodium benzoate, Ammonaps^a^ or Pheburane^a^ or Ravicti^a^	81%	17	
Number of substances among use of sodium benzonate, Ammonaps^a^, Pheburane^a^ or Ravicti^a^ (*N* = 17)			
Only one substance	38%	7	
Two substances	38%	6	
Three substances	25%	4	
Individual use of different pharmaceuticals (*N* = 21), multiple response apply			
Sodium benzoate	71%	15	
Ammonaps^a^ or Pheburane^a^	43%	9	
Ravicti^a^	33%	7	
Baclofen	14%	3	
Pegzilarginase	19%	4	
Other	33%	7	
Don't know	14%	3	

^a^ Ravicti = *glycerol phenylbutyrate*, Ammonaps or Pheburane = sodium phenylbutyrate.

All but one patient (33 years old) had a special diet prescription and 71% consumed essential amino acid (EAA) supplementation regularly. For diet adherence, 80% could follow their diet guidelines at most or all of their meals, but 45% found it difficult to follow the diet. Problem with swallowing was found among 5% of patients, and almost a fourth (24%) had problems with self‐feeding.

A majority (81%) of patients had used scavengers (sodium benzoate, sodium phenylbutyrate and/or glycerol phenylbutyrate) during the last 12 months, where 38% had used one substance, 38% had used two substances and 25% had used three substances. The reported use of three different scavengers may be due to switch during the last 12 month, as combination therapy of more than two different scavengers is not expected. Pegzilarginase was used by four patients (19%) under early access schemes.

### 3.4 Caregiver burden

Among caregivers responding to caregiver burden (*n* = 16), there were seven (44%) who had reduced or stopped working due to caregiving for the patient with ARG1‐D. Of these seven, three (43%) had more than one child with ARG1‐D. Two caregivers' (12.5%) main occupation was as carer for the child with ARG1‐D. Except for the caregivers who had stopped working, there was an additional six caregivers (38%) who did not work, and it remained unclear whether their situation stemmed from caregiving responsibilities or standard unemployment. Four caregivers (25%) reported that the patient was receiving professional assistance, and one of them had reduced or stopped working.

**FIGURE 3:**
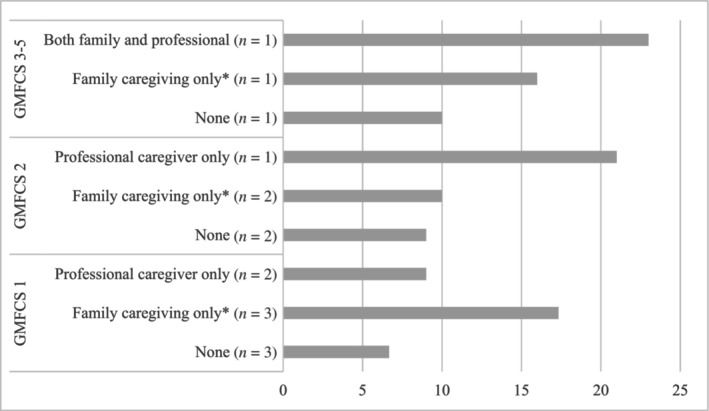
Zarit score by caregiving and GMFCS. *Family caregiver stopped/reduced working regular job.

Seven caregivers (44%) reported no to mild burden (score 0–9) on the ZBI, six caregivers (37.5%) reported mild to moderate burden (score 10–20) and three caregivers (18.5%) reported a high burden (score ≥ 20). There was a tendency for a higher caregiver burden among caregivers that had stopped/reduced working and/or that reported that the patient received professional assistance (Figure 3). The statements most caregivers agreed with in the ZBI included ‘not having enough time for yourself’, ‘feeling stressed between caring and trying to meet other responsibilities’, and ‘feel you should be doing more for your relative’.

## 4 DISCUSSION

This study was a descriptive cross‐sectional, multicentre, observational survey of patients with ARG1‐D and their caregivers in four European countries. To our knowledge, this is the first survey among this patient population, allowing for collection of subjectively reported data on aspects such as symptom severity, treatment adherence, and caregiver burden.

The study shows that there is a large variation in symptom severity among patients, varying from mild to severe mobility impairment and/or cognitive deficiency. Patients who had been diagnosed by NBS (*n* = 6) appeared to have milder symptoms, although early treatment with current options did not fully halt progression. Similar to previous research,^5^, ^10^, ^13^ this study shows that there is a delay from first symptoms to diagnosis among patients not diagnosed through NBS, and cases being misdiagnosed with a different neurological condition before their ARG1‐D diagnosis.

Reported disease management was mostly in line with management guidelines, including multidisciplinary teams with metabolic specialist, dietitian, neurologist, and physiotherapist providing care, and protein restricted diet with EAA supplementation and symptomatic treatment of disease sequalae, including nitrogen scavengers, being the most common prescribed treatments. Self‐reported adherence to the special diet was considered high but many patients found it difficult to follow the recommendations.

A significant share of caregivers reported having stopped or reduced working due to caregiving for the patient with ARG1‐D. The amount of caregiving and caregiver burden was not closely related to symptom severity. One explanation for this could be that the regulatory requirements for receiving professional assistance vary by country, and patients with the same symptom severity of disease may not be eligible for assistance in every country. Also, in our data, a subsample of caregivers with more than one child with ARG1‐D did only report the symptom severity level for their youngest child, and the symptom severity of their older child(ren) may differ.

Self‐reported data from patients and their caregivers provide valuable insights into how the patients perceive their disease and associated problems. However, symptom severity may not be as objectively measured as when reported by physicians. Symptom severity was missing for a subsample of patients since caregivers did not respond to Part II where these questions were included. The GMFCS level was therefore interpreted based on other relevant reported data for three patients. Another limitation is the use of a study‐specific instrument for measuring cognitive deficiency as standardized and validated instruments were considered too lengthy to be suitable for inclusion into a web‐based questionnaire. However, the questions were based on other validated instruments and discussed with clinical experts. Further, the cross‐sectional design did only allow for disease development to be described based on the questions about specific events that could be reported by individuals retrospectively. A prospective survey could have allowed for more details on how symptoms and caregiver burden develop over time.

The study included a small sample of patients because of the rarity of ARG1‐D. This means that individual responses may have a large impact on mean results. The small sample also implied that it was not meaningful to perform any hypothesis testing. Furthermore, less than 50 percent of the invited patients participated in the survey. Hence, due to these factors, results should be interpreted with caution. Another limitation of the study is the lack of information on plasma arginine levels, which could have contributed to further insights into the disease. However, the effect of exposure of arginine levels (e.g., long‐term exposure versus episodes of spike levels) on clinical outcomes would have required a different study design to provide valid data. Cross‐sectional data on arginine levels would not provide insights to the historical exposure for the individual patient.

Comparing the characteristics of the study sample to other studies of individuals with ARG1‐D, shows that the median age is similar (17 vs. 15,^4^ 16^10^ and 11^19^), whereas the share of females is higher (76% vs. 43.3%^5^ and 40.6%^19^). The mean age at diagnosis was similar to or lower compared to what has been reported in other studies (3.8 vs. 3.3,^19^ 6.4,^5^ and 8.8^10^). Other studies also report similar rates of spasticity (57% vs. 66%^19^ and 69%^5^), seizures (29% vs. 34%^19^ and 50%^5^), and cognitive deficiency (62% vs. 55%^5^). Mobility impairment, as reported by the GMFCS, was higher in this study compared to the baseline level in a clinical trial^19^ (1: 42% vs. 43%, 2: 26% vs. 40%, 3–5: 31% vs. 16%).

In conclusion, this study finds a significant variation in symptom severity among patients with ARG1‐D, with those diagnosed with NBS displaying milder symptoms. However, a large share of patients experiences severe cognitive and mobility deficiency despite disease management according to current management guidelines and adherence to previously available therapeutic options. In line with the result of previous research,^5^, ^10^ this suggests an unmet need among this patient population. The introduction of disease‐modifying therapies such as pegzilarginase and inclusion of the disease in NBS panels in additional countries could possibly address this need, resulting in a lower disease burden associated with ARG1‐D in the future.

## AUTHOR CONTRIBUTIONS

SO, PL, LJ, MR and JW participated in the survey design and preparation of study protocol. KMS and JBA reviewed and commented the survey and study protocol. KMS, JBA, MLC, ELT were involved in the ethical approval process in their countries and the patient recruitment. SL and SO analysed the data in collaboration with LJ, JW and MR. All authors discussed the results and approved the final manuscript.

## FUNDING INFORMATION

The study was funded by Immedica Pharma AB, Stockholm, Sweden.

## CONFLICT OF INTEREST STATEMENT

Lena Jacobson, Julia Widén and Mattias Rudebeck are employees and shareholders at Immedica Pharma AB. Karolina M Stepien has received consulting fees from Immedica Pharma AB. Jean‐Baptiste Arnoux has received consulting fees and honaria from Immedica Pharma AB. Elisa Leão Teles has received grants from Aeglia to perform clinical trials. Maria‐Luz Couce has received support from Immedica Pharma AB for participating in manuscript preparations. Sara Olofsson, Sofia Löfvendahl, and Peter Lindgren are employees at IHE which received funding for performing the study and for manuscript preparations.

## ETHICS STATEMENT

The study was approved by the Swedish Ethical Review Authority (Dnr 2022‐00363‐01), the Leeds East Research Ethics Committee in the UK (REC reference 23/YH/0023, IRAS project ID 316363), the Committee for Personal Protection South Mediterranean II in France (223 CO3), the Santiago‐Lugo Ethics Committee in Spain (2022/325), and Centro Hospitalar São João Ethics Committee in Portugal (EO 19‐22). Informed consent was obtained from all participants in the electronic survey form before any data collection.

## INFORMED CONSENT

Informed consent was obtained from all patients for being included in the study.

## ANIMAL RIGHTS

This article does not contain any studies with animal subjects.

## Supporting information


**Data S1.** Supporting information.

**Data S2.** Supporting information.

## Data Availability

Data not available due to ethical restrictions.
