# Supplementary material for: Disease burden among patients with Arginase 1 deficiency and their caregivers: A multinational, cross‐sectional survey
Source: JIMD Rep. 2024 Oct 29;65(6):450–60. doi: 10.1002/jmd2.12456 (PMC11540579; doi:10.1002/jmd2.12456)
Supplement: Supplementary file 1 — Data S1. Supporting information. [file JMD2-65-450-s002.pdf]

Patient Information Sheet and Informed Consent (For all parents and caregivers) A Survey of Resource Use and Health-Related Quality of Life in People with Arginase 1 Deficiency

We wish to ask you if you would like to participate in a research project (an online survey). Below you can find information about the project and what it means for you to participate.

Who are we and why are we asking to you take part?

Immedica Pharma AB is an independent pharmaceutical company with headquarters in Stockholm, Sweden. We're asking you to take part in this study as we are collecting information on how arginase 1 deficiency (ARG1-D) impacts the person with ARG1-D and your day-to-day life as their primary caregiver.

Invitation and Brief Summary

The purpose of this study is to find out how ARG1-D impacts resource use (for example use of health care and caregiving) and quality of life of individuals with ARG1-D and their caregivers. You are a parent or caregiver of one of around 30 patients in different European countries who have been identified to participate in the study by clinicians treating ARG1-D patients. The study is performed by the Swedish Institute for Health Economics (IHE) in collaboration with Immedica Pharma AB.

What would taking part involve?

This survey asks questions about yourself and the person with ARG1-D in terms of your backgrounds, resource use (hospital and GP visits, caregiving) related to ARG1-D and quality of life. As a parent or caregiver of someone with ARG1-D you will fall into one of three categories:

1. You are a parent/caregiver of an adult over 16 years old with ARG1-D that can answer their part of the survey by themselves. If this is the case, the person with ARG1-D has already responded to questions about themselves in Part I of the questionnaire. In this part, Part II, you will respond to your own questionnaire as the primary caregiver.

2. You are a parent/caregiver of an adult over 16 years old with ARG1-D that is unable to fill in the survey on their own behalf and will therefore require someone to assist them or answer on their behalf. Before the survey begins, you will be directed to another ‘Patient Information Sheet’ that you should read or explain to them prior to starting the survey as well as another consent that is specifically for this category of carer. This survey asks questions about their background, resource use (GP and hospital visits, caregiving) related to their ARG1-D diagnosis and their quality of life. This part of the questionnaire will take around 20 minutes to complete and please work with the person with ARG1-D as much as possible to fill in this section of the survey. You can pause the survey any time you like and finish it later, as long as you enter the survey from the same device. You can also delete or change your answers at any time if you have not already submitted your answers. You will then continue to Part 2 of the questionnaire which is designed to ask questions around your own role as primary caregiver.

3. You are a parent/caregiver of a child younger than 16 years old and will therefore need to fill in the survey on their behalf. Before the survey begins, you will be directed to an age appropriate ‘Patient Information Sheet’ that you should read or explain to them prior to starting the survey as well as another consent that is specifically for this category of carer. This survey asks questions about their background, resource use (GP and hospital visits, caregiving) related to their ARG1-D diagnosis and their quality of life. This part of the questionnaire will take around 20 minutes to complete and please work with your child as much as possible to fill in this section of the survey. You can pause the survey any time you like and finish it later, as long as you enter the survey from the same device. You can also delete or change your answers at any time if you have not already submitted your answers. You will then continue to Part 2 of the questionnaire which is designed to ask questions around your own role as primary caregiver.

As most caregivers are parents of people with ARG1-D, all questions in the survey are phrased from a parent’s point of view although we appreciate some caregivers might not be the parent or blood relative.

What are the possible benefits of taking part?

There are no direct benefits to yourself or your child or relative; however, research into such a rare disease like ARG1-D does deliver wider benefits to others with a similar condition and can help support future research into the disease. This survey may help shed light on the burden of living with ARG1-D on families and patients.

What are the possible disadvantages and risks of taking part?

Filling in the questionnaire takes a bit of time (approximately 20 minutes). As we are asking some personal questions about their health, there is the small possibility that some questions can cause upset. If they do, please speak to your child’s or relative’s doctor or nurse about this.

We ask you to answer for your youngest child with Arginase 1 Deficiency in this part of the questionnaire. When you reach the end of the survey you will receive a link to answer the questions about your other child/children.

B1. Do you care for more than one child with Arginase 1 Deficiency?

Yes ☐

No ☐

B2. Please specify how many children with Arginase 1 Deficiency you have.

☐☐☐☐☐☐☐☐☐☐

B3. Please specify the ages of your children with Arginase 1 Deficiency.

child 1 ☐☐☐☐☐☐☐☐☐☐

child 2 ☐☐☐☐☐☐☐☐☐☐

child 3 ☐☐☐☐☐☐☐☐☐☐

B4. Will your child(ren) with Arginase 1 Deficiency respond to this questionnaire by him/herself/themself?

	Yes	No
Child 1, {q2b_1.shown} years old	☐	☐
Child 2, {q2b_2.shown} years old	☐	☐
Child 3, {q2b_3.shown} years old	☐	☐

B5. if(q2a > 5, min(q2b_1.NAOK, q2b_2.NAOK, q2b_3.NAOK), if(q2a == 5, min(q2b_1.NAOK, q2b_2.NAOK, q2b_3.NAOK), if(q2a == 4, min(q2b_1.NAOK, q2b_2.NAOK, q2b_3.NAOK), if(q2a == 3, min(q2b_1.NAOK, q2b_2.NAOK, q2b_3.NAOK), if(q2a == 2, min(q2b_1.NAOK, q2b_2.NAOK), if(q2a == 1, min(q2b_1.NAOK)))))))

C1. What is your child's age today?

☐☐

Patient Information Sheet for Children 0-7 years of age A Survey of Resource Use and Health-Related Quality of Life in People with Arginase 1 Deficiency What is a Study?

A study is what you do when you want to learn about something or find out something new.

Why is this study being done?

We are doing this study to find out how your illness affects people like yourself and how it affects your mum and dad or whomever takes care of you. For example, how you feel, how often you visit the hospital or how many tests you need.

Why are you asking me?

We are asking many of the children in the country who have the same illness as you if they mind answering a few questions, with the help of your mum and dad or whoever takes care of you.

What do I have to do?

We are asking you to say 'Yes' or 'No' to participating in the study. The study only involves questions about your illness and does not involve taking any extra pills or having any extra tests with your doctor or nurse. If you say yes, we will ask you and your mum, dad or whomever takes care of you to respond to questions about you on their computer, phone or tablet.

Do I have to say yes?

No, you don't. If you decide to say no, nobody will mind and your usual care will not change in any way. If you don't want to participate in the study, all you have to do is tell your parents or whomever takes care of you.

What do I do now?

Talk with your family about what you are being asked to do. The doctors and nurses would also be happy to help you if you would like to talk to them.

Patient Information Sheet for Children 8-11 years of age A Survey of Resource Use and Health-Related Quality of Life in People with Arginase 1 Deficiency Introduction and why we need your help?

Research is done to find out more about something. Medical research sometimes involves asking people with a condition or illness some questions to find out new things about how they feel or how challenging some parts of their life may be. To do this we have prepared a survey around your illness (arginase 1 deficiency) that will take around 20 minutes to fill out with the help of yourself and your parents or whomever takes care of you. We are asking doctors in several European countries to ask their patients who have this condition if they mind participating in this study.

Will I need extra tests?

No. This is only an online survey involving a series of questions.

Do I have to take part?

No, you don't. If you decide to say no, nobody will mind and your usual care will not change in any way. If you don't want to participate in the study, all you have to do is tell your parents or caregiver.

What will happen if I say yes?

Once you and your parents or caregiver read this information sheet, they can then indicate in the consent form that you and they are happy to answer the questions in the survey. Once you are finished, the survey can be submitted and that is the end of your participation. At the end of the study, we will send a summary of the study's results to your doctor who can share this with you and your parents/caregiver. The study's results may also be published in a scientific paper. The answers you and your parents/caregiver provide will not be tied to your name or be in any way connected to you directly.

Will the information about me be kept secret?

All the information that we collect about you will be kept secret. Your survey will be given a special number so no one knows who the answers belong to.

D2. Informed Consent For parents of children under 16 year of age) A Survey of Resource Use and Health-Related Quality of Life in People with Arginase 1 Deficiency (ARG1-D) Informed Consent for Parents and Caregivers on Behalf of Children Less than 16 years of age

Before you start answering the questions, we ask you to give your formal consent to participate in the "Survey of Resource Use and Health-Related Quality of Life in People with arginase 1 deficiency (ARG1-D)" on behalf of your child/the person with ARG1-D.

By clicking the box below I certify that:

I have read the age appropriate patient information sheet out loud to my child with ARG1-D and, to the best of my understanding, he/she grants assent to participate in this study. In my opinion, they would have no objection to taking part in the above study. I consent to my responses being treated as described in the above Patient Information Sheet. I understand that the person with ARG1-D or myself cannot and will not be identified in any future publication or report that is based on my data. I understand that my participation is voluntary and that I can choose not to participate without giving any reason. This will in no way affect my child's medical care or legal rights. I am aware that I can ask for more information from the contact persons for the study or from the patient's clinician.

I give my informed consent to take part in the study:

Yes ☐

No ☐

Patient Information Sheet for Adults unable to respond for themselves A Survey of Resource Use and Health-Related Quality of Life in People with Arginase 1 Deficiency Introduction and why we need your help?

Research is done to find out more about a specific topic. Medical research sometimes involves asking people with a condition or illness some questions to find out new things about how they feel or how challenging some parts of their life may be. To do this we have prepared a survey around your illness (arginase 1 deficiency) that will take around 20 minutes to fill out with the help of yourself and your parents (or whoever looks after you). We are asking doctors in several European countries to ask their patients who have this condition if they mind participating in this study.

Will I need extra tests?

No. This is only an on-line survey involving a series of questions.

Do I have to take part?

No, you don't. If you decide to say no, nobody will mind and your usual care will not change in any way. If you don't want to participate in the study, all you have to do is tell your parents or caregiver.

What will happen if I say yes?

Once you and your parents or main caregiver read this information sheet, they can then indicate on the consent form that you and they are happy to answer the questions in the survey. Once you are finished, the survey can be submitted and that is the end of your participation. At the end of the study, we will send a summary of the study's results to your doctor who can share this with you and your parents or main caregiver. The study's results may also be published in a scientific paper. The answers you and your parents or main caregiver provide will not be tied to your name or be in any way connected to you directly.

Will the information about me be kept secret?

All the information that we collect about you will be kept secret. Your survey will be given a special number so no-one knows who they belong to.

What happens if I change my mind?

You can change your mind at any time before you or your parents or main caregiver submit the survey.

If you have any questions or worries about the information in this leaflet or anything else, please ask your parent or caregiver to speak to one of your nurses or doctors

UCLH: Consultant: Dr Elaine Murphy Telephone: 0203 448 83603 Email: elaine.murphy8@nhs.net

Research Nurse: Tiggy Beyene Telephone: 07976691121 Email: tiggy.beyene@nhs.net

Salford: Consultant: Karolina Stepien Telephone: 0161 2064365 Email: karolina.stepien@nca.nhs.uk

Research Nurse: Marie Meehan (study coordinator) Email: marie.meehan@nca.nhs.uk Telephone:_0161 2064365

GOSH Consultant: Dr. Spyros Batzios Telephone: 02074059200 extension 5539 Email: Spyros.Batzios@gosh.nhs.uk

Bradford Consultant: Dr Arunabha Ghosh Telephone : 0161 7012137 Email: arunabha.ghosh@mft.nhs.uk

Consultant: Dr Sergei Korenev Telephone: 0161 7012137 Email: sergei.korenev@mft.nhs.uk

or contact IHE at: Sara Olofsson (sara.olofsson@ihe.se, research manager IHE)

Patient Information Sheet and Informed Consent (For parents and caregivers of adults unable to respond for themselves: Consultee)

Your healthcare care professional feels your child/relative is unable to decide for themselves whether to participate in this research (an on-line questionnaire).

Patient Information Sheet for Children 0-7 years of age A Survey of Resource Use and Health-Related Quality of Life in People with Arginase 1 Deficiency What is a Study?

A study is what you do when you want to learn about something or find out something new.

Why is this study being done?

We are doing this study to find out how your illness affects people like yourself and how it affects your mum and dad or whomever takes care of you. For example, how you feel, how often you visit the hospital or how many tests you need.

Why are you asking me?

We are asking many of the children in the country who have the same illness as you if they mind answering a few questions, with the help of your mum and dad or whoever takes care of you.

What do I have to do?

We are asking you to say 'Yes' or 'No' to participating in the study. The study only involves questions about your illness and does not involve taking any extra pills or having any extra tests with your doctor or nurse. If you say yes, we will ask you and your mum, dad or whomever takes care of you to respond to questions about you on their computer, phone or tablet.

Do I have to say yes?

No, you don't. If you decide to say no, nobody will mind and your usual care will not change in any way. If you don't want to participate in the study, all you have to do is tell your parents or whomever takes care of you.

What do I do now?

Talk with your family about what you are being asked to do. The doctors and nurses would also be happy to help you if you would like to talk to them.

Patient Information Sheet for Children 8-11 years of age A Survey of Resource Use and Health-Related Quality of Life in People with Arginase 1 Deficiency Introduction and why we need your help?

Research is done to find out more about something. Medical research sometimes involves asking people with a condition or illness some questions to find out new things about how they feel or how challenging some parts of their life may be. To do this we have prepared a survey around your illness (arginase 1 deficiency) that will take around 20 minutes to fill out with the help of yourself and your parents or whomever takes care of you. We are asking doctors in several European countries to ask their patients who have this condition if they mind participating in this study.

Will I need extra tests?

No. This is only an online survey involving a series of questions.

Do I have to take part?

No, you don't. If you decide to say no, nobody will mind and your usual care will not change in any way. If you don't want to participate in the study, all you have to do is tell your parents or caregiver.

What will happen if I say yes?

Once you and your parents or caregiver read this information sheet, they can then indicate in the consent form that you and they are happy to answer the questions in the survey. Once you are finished, the survey can be submitted and that is the end of your participation. At the end of the study, we will send a summary of the study's results to your doctor who can share this with you and your parents/caregiver. The study's results may also be published in a scientific paper. The answers you and your parents/caregiver provide will not be tied to your name or be in any way connected to you directly.

Will the information about me be kept secret?

All the information that we collect about you will be kept secret. Your survey will be given a special number so no one knows who the answers belong to.

G1. What is your child's age today?

☐☐

G2. What is your child's biological gender?

- Female ☐
- Male ☐

G3. What is your child's current living arrangement?

- Living with parent(s) ☐
- Live in own home ☐
- Supported living services for people with disabilities ☐
- Other, please specify ☐

Other, please specify

G4. How many adults (18 years and above) does your child live with?

- 0 ☐
- 1 ☐
- 2 ☐
- 3 ☐
- 4 ☐
- 5 ☐
- more than 5 ☐

G5. How many children (below 18 years) does your child live with?

- 0 ☐
- 1 ☐
- 2 ☐
- 3 ☐
- 4 ☐
- 5 ☐
- more than 5 ☐

G6. Is your child in school or what is his/her current main occupation?

- Below school age ☐
- Primary/secondary school ☐
- Student/University ☐
- Employed or self-employed ☐
- Sick-leave or early retirement ☐
- Unemployed or looking for work ☐
- Other, please specify: ☐

Other, please specify:

G7. What is the highest level of education your child has completed?

- Did not complete primary school ☐
- Primary school ☐
- Secondary school ☐
- University ☐
- Don't know ☐
- Other, please specify ☐

Other, please specify

G8. **Does or has your child received specialised education due to his/her Arginase 1 Deficiency diagnosis?**

No ☐

Don’t know ☐

Yes, please specify ☐

Yes, please specify

Section 2 of 11. Disease and symptoms

H1. **At what age did your child experience his/her first symptom of Arginase 1 Deficiency?**

Don’t know ☐

Age: ☐

Age:

H2. **At what age did you notice the first problem in your child's movement abilities due to Arginase 1 Deficiency?**

No problems with regards to movement ☐

Don't know ☐

Age: ☐

Age:

H3. **At what age did you notice the first problem in your child's ability to think, read, learn, remember, reason, or pay attention?**

No problems ☐

Don't know ☐

Age: ☐

Age:

H4. **What was the first problem(s) you noticed related to your childs ability to think, read, learn, remember, reason, or pay attention?**

H5. **At what age was your child diagnosed with Arginase 1 Deficiency?**

Diagnosed by new-born screening ☐

Don't know ☐

Age: ☐

Age:

H6. **Did your child receive an incorrect diagnosis before receiving his/her Arginase 1 Deficiency diagnose?**

Yes ☐

No ☐

Don't know ☐

H7. **What diagnosis did your child receive first?**

H8. **At what age did your child receive this incorrect diagnosis?**

Don’t know ☐

Age: ☐

Age:

H9. **Has your child received a liver transplant?**

Yes ☐

No ☐

H10. **Does your child experience spasticity (stiffness of muscle, which might interfere with movement, speech, or be associated with discomfort or pain) due to Arginase 1 Deficiency? Check all that apply.**

Yes, lower limbs (legs) ☐

Yes, upper limbs (arms) ☐

No ☐

Don’t know ☐

H11. **Has your child experienced seizures due to Arginase 1 Deficiency? Seizures are bursts of electrical activity in the brain that temporarily affect how it works.**

Yes ☐

No ☐

Don’t know ☐

H12. How many seizures has your child experienced during the last 4 weeks?

- Don’t know ☐
- Number of seizures: ☐

Number of seizures:

H13. Is your child taking any medication to help control seizures (i.e. anti-epileptic drug)?

- Yes ☐
- No ☐
- Don’t know ☐

H14. Has your child experienced any other symptoms due to Arginase 1 Deficiency?

- Yes ☐
- No ☐
- Don’t know ☐

H15. Please specify your child's other symptoms:

H16. Does your child have any other long-term (6 months or more) illness or disability?

- Yes ☐
- No ☐
- Prefer not to say ☐

H17. What other illness/disability does your child have? Check all that apply.

- Hypertension (high blood pressure) ☐
- Diabetes type 2 ☐
- Cerebral palsy ☐

Anaemia (lower than normal number of red blood cells) ☐

Kidney disease ☐

Swallowing problems ☐

Low body weight/Eating disorder ☐

Thyroid disease ☐

Heart problems ☐

Lung diseases ☐

Muscle and bone disorder ☐

Chronic obstructive pulmonary disease (COPD) ☐

Stroke ☐

Restless legs syndrome ☐

Chronic heart failure ☐

Gastro-oesophageal reflux (relating to increased stomach acid) ☐

Depression ☐

None of the above ☐

Section 3 of 11. Health care use

I1. During the last 12 months, has your child visited the A&E (emergency) department at the hospital due to Arginase 1 Deficiency?

Yes ☐

No ☐

Don't know ☐

I2. How many times has your child visited the A&E (emergency) department at the hospital due to Arginase 1 Deficiency during the last 12 months?

Don't know ☐

Number of times: ☐

Number of times:

I3. **During the last 12 months, has your child been hospitalised due to Arginase 1 Deficiency?**

Yes ☐

No ☐

Don't know ☐

I4. **How many times has your child been hospitalised due to Arginase 1 Deficiency during the last 12 months?**

☐☐☐☐☐☐☐☐☐☐

I5. **For how many days was your child hospitalised?1st hospitalisation:**

days ☐

I6. **For how many days was your child hospitalised?2nd hospitalisation:**

days ☐

I7. **For how many days was your child hospitalised?3rd hospitalisation**

days ☐

I8. **For how many days was your child hospitalised?4th hospitalization**

days ☐

I9. **For how many days was your child hospitalised?5th hospitalization**

days ☐

I10. **For how many days was your child hospitalised?6th hospitalization**

days ☐

I11. **For how many days was your child hospitalised?7th hospitalization**

days ☐

I12. **For how many days was your child hospitalised?8th hospitalization**

days ☐

I13. **For how many days was your child hospitalised?9th hospitalization**

days ☐

I14. **For how many days was your child hospitalised?10th hospitalization**

days ☐

I15. **For how many days was your child hospitalised?11th hospitalization**

days ☐

I16. **For how many days was your child hospitalised?12th hospitalization**

days ☐

I17. During the last 12 months, has your child visited any of the below health care staff due to Arginase 1 Deficiency? Do not include visits to the A&E or hospitalization that you have responded to before, check all that apply.

- General practitioner ☐
- Neurologist ☐
- Paediatrician ☐
- Metabolic specialist ☐
- Nurse ☐
- Physiotherapist or rehabilitation specialist ☐
- Occupational therapist ☐
- Psychologist ☐
- Dietitian ☐
- Geneticist ☐
- Speech and language therapist ☐
- None of the above ☐
- Other, please specify: ☐

Other, please specify:

I18. How many visits to each of the following has your child done in the last 12 months?General practitioner

Number of visits during the last 12 months

I19. How many visits to each of the following has your child done in the last 12 months?Neurologist

Number of visits during the last 12 months

I20. How many visits to each of the following has your child done in the last 12 months?Paediatrician

Number of visits during the last 12 months

I21. How many visits to each of the following has your child done in the last 12 months?Metabolic specialist

Number of visits during the last 12 months

I22. **How many visits to each of the following has your child done in the last 12 months?Nurse**

Number of visits during the last 12 months [ ]

I23. **How many visits to each of the following has your child done in the last 12 months?Physiotherapist or rehabilitation specialist**

Number of visits during the last 12 months [ ]

I24. **How many visits to each of the following has your child done in the last 12 months?Occupational therapist**

Number of visits during the last 12 months [ ]

I25. **How many visits to each of the following has your child done in the last 12 months?Psychologist**

Number of visits during the last 12 months [ ]

I26. **How many visits to each of the following has your child done in the last 12 months?Dietitian**

Number of visits during the last 12 months [ ]

I27. **How many visits to each of the following has your child done in the last 12 months?Geneticist**

Number of visits during the last 12 months [ ]

I28. **How many visits to each of the following has your child done in the last 12 months?Speech and language therapist**

Number of visits during the last 12 months [ ]

I29. **How many visits to each of the following has your child done in the last 12 months?Other {q34_other}**

Number of visits during the last 12 months [ ]

I30. **During the last 12 months, has your child had any of the below test due to Arginase 1 Deficiency? Check all that apply.**

- [ ] Blood test
- [ ] Ultrasound
- [ ] Electrocardiogram (ECG)Test to evaluate the heart
- [ ] Electroencephalogram (EEG)Test to evaluate the brain/ seizure activity
- [ ] Magnetic resonance imaging (MRI)
- [ ] Computed tomography (CT) scan
- [ ] Dont know

Other, please specify:

Other, please specify:

I31. How many tests has your child done during the last 12 months?Blood test

Number of tests during the last 12 months

I32. How many tests has your child done during the last 12 months?Ultrasound

Number of tests during the last 12 months

I33. How many tests has your child done during the last 12 months?Electrocardiogram (ECG)Test to evaluate the heart

Number of tests during the last 12 months

I34. How many tests has your child done during the last 12 months?Electroencephalogram (EEG)Test to evaluate the brain/ seizure activity

Number of tests during the last 12 months

I35. How many tests has your child done during the last 12 months?Magnetic resonance imaging (MRI) scan

Number of tests during the last 12 months

I36. How many tests has your child done during the last 12 months?Computed tomography (CT) scan

Number of tests during the last 12 months

I37. How many tests has your child done during the last 12 months?Other; {q35_other}

Number of tests during the last 12 months

I38. During the last 12 months, has your child been treated with injections of botulinum toxin (Botox, Xeomin or Dysport) for muscle stiffness?

- Yes
- No
- Don’t know

I39. **How many times has your child received this treatment in the last 12 months?**

Don't know ☐

Number of times: ☐

Number of times:

I40. **During the last 12 months, has your child received surgical treatments for muscle stiffness?**

Yes ☐

No ☐

Don't know ☐

I41. **How many surgical treatments have your child received in the last 12 months?**

Don't know ☐

Number of treatments: ☐

Number of treatments:

Section 4 of 11. Diet and medications

J1. **Has your child been prescribed a special diet for treatment of his/her Arginase 1 Deficiency?**

Yes ☐

No ☐

Don’t know ☐

J2. **In the past 7 days, did your child manage to adhere to this prescribed diet for his/her Arginase 1 Deficiency?**

- Yes, all the meals ☐
- Most of the time ☐
- Sometimes ☐
- A little of the time ☐
- No, none of the meals ☐
- Don't know ☐

J3. **How easy/difficult does your child think it is to follow the dietician's and/or physician's guidelines for his/her Arginase 1 Deficiency diet?**

- Very easy ☐
- Easy ☐
- Difficult ☐
- Very difficult ☐
- Don't know ☐

J4. **Does your child have problems with swallowing and/or self-feed? Check all that apply.**

- No, can self-fed with no problems ☐
- Yes, swallowing ☐
- Yes, self-feed ☐
- Don't know ☐

J5. **Does your child regularly consume protein substitute? By protein substitute we mean an amino acid mixture prescribed by your child's physician/dietician.**

- Yes ☐
- No ☐
- Don't know ☐

J6. **During the last 12 months, has your child used any of the medicines listed below for his/her Arginase 1 Deficiency? Check all that apply.**

- Sodium benzoate ☐
- Sodium phenylbutyrate (Ammonaps or Pheburane) ☐
- Glycerol phenylbutyrate (Ravicti) ☐
- Baclofen (muscle relaxant medication for spasticity) ☐

Pegzilarginase ☐

None of the above ☐

Other: ☐

Other:

J7.

Section 5 of 11. Professional Caregiving and Assistance

K1. During the last 12 months, has your child received professional assistance as a consequence of Arginase 1 Deficiency? Do not include assistance/caregiving by relatives or friends.

Yes ☐

No ☐

Don’t know ☐

K2. What type of professional assistance has your child received? Check all that apply.

Daily activities (leisure, school, work) ☐

Personal care ☐

Household ☐

Transportation ☐

Other, please specify: ☐

Other, please specify:

K3. **How many hours per week does your child receive professional assistance in total?**

Don't know ☐

hours of assistance per week ☐

hours of assistance per week

K4. **At what age did your child start receiving assistance?**

Don't know ☐

age ☐

age

K5. **Have you made any adjustments in your home as a consequence of your child's Arginase 1 Deficiency?**

Yes ☐

No ☐

Don't know ☐

K6. **What type of adjustments have you made?**

K7. **Does your child use any mobility aids or devices due to Arginase 1 Deficiency?**

Yes ☐

No ☐

Don't know ☐

K8. **What type of mobility aid does your child use? Check all that apply.**

Wheelchair ☐

Walking aids (walker, rollator, crutches, canes) ☐

Walking stabilators (casts, splints etc.) ☐

Other, please specify ☐

Other, please specify

K9. **At what age did your child start using the mobility aid or devices?Wheelchair**

Age when started using

K10. **At what age did your child start using the mobility aid or devices?Walking aids (walker, rollator, crutches, canes)**

Age when started using

K11. **At what age did your child start using the mobility aid or devices?Walking stabilators (casts, splints etc.)**

Age when started using

K12. **At what age did your child start using the mobility aid or devices?Other, {q47b_other}**

Age when started using

Section 6 of 11. Work

L1. **Is your child employed or self-employed?**

Yes ☐

No ☐

Don’t know ☐

L2. **Is your child fully employed?**

Yes ☐

Don't know ☐

No, specify the % that your child works: ☐

No, specify the % that your child works:

L3. **Is your child on long-term sick-leave or on early retirement due to Arginase 1 Deficiency? Check all that apply.**

Yes – sick-leave full-time ☐

Comment

Yes – sick-leave part-time– please specify percentage ☐

Comment

Yes - early retirement full-time ☐

Comment

Yes – early retirement part-time – please specify percentage ☐

Comment

No ☐

Comment

Don't know ☐

Comment

L4. **At what age did your child go on sick-leave/early retirement?**

Don’t know ☐

Age: ☐

Age:

L5. **Has your child been absent from work due to symptoms from Arginase 1 Deficiency during the last 4 weeks?**

Yes ☐

No ☐

Don’t know ☐

L6. **How many hours has he/she been absent from work due to symptoms from Arginase 1 Deficiency during the last 4 weeks?**

Don't know ☐

In total hours, around ☐

In total hours, around

L7. **During the past seven days, how much did your child's health problems affect his/her productivity while he/she was working?**

Think about days your child was limited in the amount or kind of work he/she could do, days your child accomplished less than he/she would like, or days your child could not do his/her work as carefully as usual. If health problems affected his/her work only a little, choose a low number. Choose a high number if health problems affected his/her work a great deal.

Consider only how much health problems affected productivity while he/she was working.

Health problems had no effect on my child's work0	1	2	3	4	5	6	7	8	9	Health problems completely prevented my child from working 10
☐	☐	☐	☐	☐	☐	☐	☐	☐	☐	☐

L8. **During the past seven days, how much did your child's health problems affect his/her ability to do regular daily activities?**

By regular activities, we mean the usual activities your child do, such as playing, exercising, studying etc. Think about times your child were limited in the amount or kind of activities your child could do and times your child accomplished less than your child would like. If health problems affected your child's activities only a little, choose a low number. Choose a high number if health problems affected his/her activities a great deal.

Consider only how much health problems affected your child's ability to do your child's regular daily activities, other than work at a job.

Health problems had no effect on my child's daily activities0	1	2	3	4	5	6	7	8	9	Health problems prevented my child from doing his/her daily activities 10
☐	☐	☐	☐	☐	☐	☐	☐	☐	☐	☐

Section 11 of 11. Quality of Life*"Please select the ONE box that you think best describes the person's health TODAY. You should not answer on behalf of the person, but rather rate the person's health as you see it."*

M1. **MOBILITY**

- No problems in walking about ☐
- Slight problems in walking about ☐
- Moderate problems in walking about ☐
- Severe problems in walking about ☐
- Unable to walk about ☐

"Please select the ONE box that you think best describes the person's health TODAY. You should not answer on behalf of the person, but rather rate the person's health as you see it."

N1. **SELF-CARE**

- No problems washing or dressing him/herself ☐
- Slight problems washing or dressing him/herself ☐
- Moderate problems washing or dressing him/herself ☐
- Severe problems washing or dressing him/herself ☐
- Unable to wash or dress him/herself ☐

"Please select the ONE box that you think best describes the person's health TODAY. You should not answer on behalf of the person, but rather rate the person's health as you see it."

O1. USUAL ACTIVITIES (e.g. work, study, housework, family or leisure activities)

No problems doing his/her usual activities ☐

Slight problems doing his/her usual activities ☐

Moderate problems doing his/her usual activities ☐

Severe problems doing his/her usual activities ☐

Unable to do his/her usual activities ☐

"Please select the ONE box that you think best describes the person's health TODAY. You should not answer on behalf of the person, but rather rate the person's health as you see it."

P1. PAIN/ DISCOMFORT

No pain or discomfort ☐

Slight pain or discomfort ☐

Moderate pain or discomfort ☐

Severe pain or discomfort ☐

Extreme pain or discomfort ☐

"Please select the ONE box that you think best describes the person's health TODAY. You should not answer on behalf of the person, but rather rate the person's health as you see it."

Q1. ANXIETY / DEPRESSION

Not anxious or depressed ☐

Slightly anxious or depressed ☐

Moderately anxious or depressed ☐

Severely anxious or depressed ☐

Extremely anxious or depressed ☐

R1.

We would like to know how good or bad you think the person's health is TODAY.

- **You will see a scale numbered from 0 to 100.**

- **100 means the best health you can imagine.**

0 means the worst health you can imagine.

- **Please indicate on the scale how you think the person's health is TODAY.**

THE PERSON'S HEALTH

TODAY =

Questionnaire Part II: Questions about the Caregiver
Section 8 of 11. Background questions

S1. What is your age today?

S2. What is your biological gender?

Female ☐
Male ☐
Prefer not to say ☐

S3. What is your relationship to the patient?

Parent ☐
Other, please specify: ☐

Other, please specify:

S4. **Do you live in the same household as the person with arginase deficiency?**

- Yes ☐
- No ☐

S5. **How many adults (18 years and above) do you live with?**

- 0 ☐
- 1 ☐
- 2 ☐
- 3 ☐
- 4 ☐
- 5 ☐
- more than 5 ☐

S6. **How many children (below 18 years) do you live with?**

- 0 ☐
- 1 ☐
- 2 ☐
- 3 ☐
- 4 ☐
- 5 ☐
- more than 5 ☐

S7. **What is the highest level of education you have completed?**

- Primary school ☐
- Secondary school ☐
- University ☐
- No formal education ☐
- Other, please specify: ☐

Other, please specify:

S8. **What is your current main occupation?**

Choose the occupation that takes most of your time.

- Student ☐
- Employed ☐
- Parental leave ☐
- Sick-leave or early retirement ☐
- Unemployed or looking for work ☐
- Retired ☐
- Registered as carer for the child with Arginase 1 Deficiency ☐

Section 9 of 11. Caregiving

T1. **Who cares for your child(ren) with Arginase 1 Deficiency in your family (do not include professional assistance)? Check all that apply.**

- Myself ☐
- My partner ☐
- Other, please specify ☐

Other, please specify

T2. **Who would you say is the primary caregiver?**

- Myself ☐
- My partner ☐
- Other, please specify ☐

Other, please specify

GMFCS Family Report Questionnaire: Children Aged 2 to

U1. My child..

- [ ] Has difficulty controlling head and trunk posture in most positionsand uses specially adapted seating to sit comfortably
and has to be lifted by another person to move about
- [ ] Can sit on own when placed on the floor and can move within a roomand uses hands for support to maintain sitting balance
and usually uses adaptive equipment for sitting and standing
and moves by rolling, creeping on somach or crawling
- [ ] Can sit on own and walk short distances with a walking aid (such as a walker, rollator, crutches, canes, etc.)and may need help from adult for steering and turning when walking with an aid
and usually sits on floor in a “W-sitting” position and may need help from an adult to get into sitting
and may pull to stand and cruise short distances
and prefers to move by creeping and crawling
- [ ] Can sit on own and usually moves by walking with a walking aidand may have difficulty with sitting balance when using both hands to play
and can get in and out of sitting positions on own
and can pull to stand and cruise holding onto furniture
and can crawl, but prefers to move by walking
- [ ] Can sit on their own and moves by walking without a walking aidand is able to balance in sitting when using both hands to play
and can move in and out of sitting and standing positions without help from an adult
and prefers to move by walking

GMFCS Family Report Questionnaire: Children Aged 4 to

V1. My child..

- [ ] Has difficulty sitting on their own and controlling their head and body posture in most positionsand has difficulty achieving any voluntary control of movement
and needs a specially-adopted supportive chair to sit comfortably
and has to be lifted or hoisted by another person to move
- [ ] Can sit on their own but does not stand or walk without significant support and adult supervisionand may need extra body / trunk support to improve arm
and hand function and usually needs adult assistance to get in and out of chair
and may achieve self-mobility using a powered wheelchair or is transported in the community
- [ ] Can walk on their own using a walking aid (such as a walker, rollator, crutches, canes, etc.)and can usually get in and out of a chair without adult assistans
and may use a wheelchair when traveling long distances or outside
and finds it difficult to climb stairs or walk on an uneven surface without considerable help
- [ ] Can walk on their own without using a walking aid, but has difficulty walking long distances or on eleven surfacesand can sit in a normal adult chair and use both hands freely
and can move from the floor to standing without adult assistance
and needs to hold the handrail when going up or down stairs and is not yet able to run and jump
- [ ] Can walk on their own without using a walking aid, including fairly long distances, outdoors and on uneven surfacesand can move from the floor or a chair to standing without using their hands for support
and can go up and down the stairs without needing to hold the handrail
and is beginning to run and jump

GMFCS Family Report Questionnaire: Children Aged 6 to

W1. My child...

- [ ] Has difficulty sitting on their own and controlling their head and body posture in most positionsand has difficulty achieving any voluntary control of movement
and needs a specially supportive chair to sit comfortably
and has to be lifted or hoisted by another person to move
- [ ] Can sit on their own but does not stand or walk without significant supportand therefore relies mostly on wheelchair at home, school
and in the community and often needs extra body / trunk support to improve arm and hand function
and may achieve self-mobility using a powered wheelchair
- [ ] Can stand on their own and only walks using a walking aid (such as a walker, rollator, crutches, canes, etc.)and fins it difficult to climb stairs or walk on
uneven surfaces
and may use a wheelchair when traveling for long distances or in crowds
- [ ] Can walk on their own without using walking aids, but needs to hold the handrail when going up or
down stairsand often finds it difficult to walk on uneven surfaces, slopes or in crowds
- [ ] Can walk on their own without using walking aids and can go up or down stairs without needing to hold the handrailand walks whenever they want to go
(including uneven surfaces, slopes or in crowds)
and can run and jump although their speed, balance, and coordination may be slightly limited

GMFCS Family Report Questionnaire: Children Aged >12 Years

Please read the following and mark only one box beside the description that best represents your child's movement abilities

X1. My child...

- ☐ Has difficulty sitting on their own and controlling their head an body posture in most positionsand has difficulty achieving any voluntary control of movement and needs a specially adopted chair to sit comfortably and be transported anywhere and has to be lifted or hoisted by another person or special equipment to move
- ☐ Can sit with some pelvic and trunk support but does not stand or walk without significant supportand therefore always relies on wheelchair when outdoors and can achieve self-mobility using a powered wheelchair and can crawl or roll to a limited extent to move around indoors
- ☐ Can stand on their own and only walks using a walking aid (such as a walker, rollator, crutches, canes, etc.)and finds it difficult to climb stairs, or walk on uneven surfaces without support and uses a variety of means to move around depending on the circumstances and prefers to use a wheelchair to travel quickly or even longer distances
- ☐ Can walk on their own without using walking aids, but needs to hold the handrail when going up or down stairsand therefore walks in most settings and often finds it difficult to walk on uneven surfaces, slopes or in crowds and may occasionally prefer to use a walking aid (such as a cane or crutch) or a wheelchair to travel quickly or over longer distances
- ☐ Can walk on their own without using walking aids, and can go up or down stairs without needing to hold the handrailand walks wherever they want to go (including uneven surfaces, slopes or in crowds) and can run and jump although their speed, balance, and coordination may be limited

Cognitive abilities, youngest child
Mark one box that best describes your child’s current cognitive abilities.

Y1. If you compare your child to other children of the same age, how would you rate your child’s ability to *communicate*

	0No problems	1Some problems	2Moderate problems	3Severe problems	4Cannot do at all	6Don't know	5 Not relevant
Understand what other children say (Receptive)	☐	☐	☐	☐	☐	☐	☐
Express needs, wants, opinions etc. (Expressive)	☐	☐	☐	☐	☐	☐	☐
Read and write	☐	☐	☐	☐	☐	☐	☐

Y2. If you compare your child to other children of the same age, how would you rate your child’s ability to *socialize*

	0No problems	1Some problems	2Moderate problems	3Severe problems	4Cannot do at all	5Don’t know	6Not relevant
Building and sustaining relationships (interpersonal relationships)	☐	☐	☐	☐	☐	☐	☐
Functioning in play and leisure (play and leisure)	☐	☐	☐	☐	☐	☐	☐
Deal with stressful situations (Coping skills)	☐	☐	☐	☐	☐	☐	☐

Y3. If you compare your child to other children of the same age, how would you rate your child’s *ability to learn, think and solve problems*

	0No problems	1Some problems	2Moderate problems	3Severe problems	4Cannot do at all	5Don’t know	6Not relevant
Concentrate	☐	☐	☐	☐	☐	☐	☐
Recall/Memorize	☐	☐	☐	☐	☐	☐	☐
Understand and relate to the concept of time	☐	☐	☐	☐	☐	☐	☐

	0No problems	1Some problems	2Moderate problems	3Severe problems	4Cannot do at all	5Don't know	6Not relevant
Perform tasks in school/work	☐	☐	☐	☐	☐	☐	☐

Y4. If you compare your child to other children of the same age, how would you rate your child's *ability to perform activities of daily living*

	0No problems	1Some problems	2Moderate problems	3Severe problems	4Cannot do at all	5Don't know	6 Not relevant
Personal care, washing and dressing yourself	☐	☐	☐	☐	☐	☐	☐
Domestic chores, for example cleaning, cooking	☐	☐	☐	☐	☐	☐	☐
Community activities (school, work)	☐	☐	☐	☐	☐	☐	☐

Z1. What type of assistance have you and others in your family provided as a consequence of Arginase 1 Deficiency (i.e. assistance that you would not provide if the child did not have Arginase 1 Deficiency? Check all that apply.

daily activities (leisure, school) ☐

personal care ☐

household ☐

transportation ☐

other ☐

don't know ☐

Z2. In the last 7 days, how many hours per day did you provide assistance to your child/children as a consequence of their Arginase 1 Deficiency?

Don't know ☐

Hours per day ☐

Hours per day

Z3. **In the last 7 days, for how many hours per day did others in your family provide assistance as a consequence of Arginase 1 Deficiency?**

Don’t know ☐

hours per day ☐

hours per day

[ ]

Z4. **In the last 7 days, what proportion of time do you estimate was spent caring for your child(ren)?**

First child {q2b_1.shown} [ ]

Second child {q2b_2.shown} [ ]

Third child {q2b_3.shown} [ ]

Section 10 of 11. Work situation

AA1. **Are you employed or self-employed? (Any level of employment or self-employment, not just main occupation).**

Yes ☐

No ☐

Don’t know ☐

AA2. **Are you fully employed?**

Yes ☐

No, specify the % that you work: ☐

No, specify the % that you work:

[ ]

AA3. **Have you reduced your employment to care for your child with Arginase-1 deficiency?**

Yes ☐

No ☐

AA4. **At what age did you reduce your employment?**

AA5. **Have you taken time off from work in the last 4 weeks due to caring for your child with Arginase-1 deficiency?**

Yes ☐
No ☐
Don't know ☐

AA6. **How many hours have you been absent from work due to caregiving for your child/children with Arginase 1 Deficiency during the last 4 weeks?**

Don't know ☐
In total hours, around ☐

In total hours, around

AA7. **Please estimate what proportion of time you spend caring for each of your children with Arginase 1 Deficiency when you're absent from work due to caregiving?**

% Child 1
% Child 2
% Child 3

AA8. **Have you stopped working because of caregiving?**

Yes ☐
No ☐

AA9. **At what age did you stop working?**

AB1. **During the past seven days, how much did your child's/children's health problems affect your productivity while you were working?**

Think about days you were limited in the amount or kind of work you could do, days you accomplished less than you would like, or days you could not do your work as carefully as usual. If the health problems of your child(ren) affected your work only a little, choose a low number. Choose a high number if the health problems of your child(ren) affected your work a great deal.

Consider only how much the health problems of your child(ren) affected productivity while you were working.

0 – my child's health problems had no effect on my work	1	2	3	4	5	6	7	8	9	10 – my child's health problems completely prevented me from working
☐	☐	☐	☐	☐	☐	☐	☐	☐	☐	☐

AB2. **During the past seven days, how much did your child's/children's health problems affect your ability to do your regular daily activities, other than work at a job?**

By regular activities, we mean the usual activities you do, such as work around the house, shopping, childcare, exercising, studying, etc. Think about times you were limited in the amount or kind of activities you could do and times you accomplished less than you would like. If the health problems of your child(ren) affected your activities only a little, choose a low number. Choose a high number if the health problems of your child(ren) affected your activities a great deal.

Consider only how much your child's/children's health problems affected your ability to do your regular daily activities, other than work at a job.

0 – my child's/children's health problems had no effect on my daily activities	1	2	3	4	5	6	7	8	9	10 – my child's/children's health problems prevented me from doing my daily activities
☐	☐	☐	☐	☐	☐	☐	☐	☐	☐	☐

Section 11 of 11. Quality of LifePlease select the ONE box that best describes your health TODAY.

AC1. MOBILITY

- I have no problems in walking about ☐
- I have slight problems in walking about ☐
- I have moderate problems in walking about ☐
- I have severe problems in walking about ☐
- I am unable to walk about ☐

Please select the ONE box that best describes your health TODAY.

AD1. SELF-CARE

- I have no problems washing or dressing myself ☐
- I have slight problems washing or dressing myself ☐
- I have moderate problems washing or dressing myself ☐
- I have severe problems washing or dressing myself ☐
- I am unable to wash or dress myself ☐

Please select the ONE box that best describes your health TODAY.

AE1. USUAL ACTIVITIES (e.g. work, study, housework, family or leisure activities)

- I have no problems doing my usual activities ☐
- I have slight problems doing my usual activities ☐
- I have moderate problems doing my usual activities ☐
- I have severe problems doing my usual activities ☐
- I am unable to do my usual activities ☐

Please select the ONE box that best describes your health TODAY.

AF1. PAIN / DISCOMFORT

- I have no pain or discomfort ☐
- I have slight pain or discomfort ☐
- I have moderate pain or discomfort ☐
- I have severe pain or discomfort ☐
- I have extreme pain or discomfort ☐

Please select the ONE box that best describes your health TODAY.

AG1. ANXIETY / DEPRESSION

- I am not anxious or depressed ☐
- I am slightly anxious or depressed ☐
- I am moderately anxious or depressed ☐
- I am severely anxious or depressed ☐
- I am extremely anxious or depressed ☐

AH1. We would like to know how good or bad your health is TODAY. This scale is numbered from 0 to 100. 100 means the <u>best</u> health you can imagine.
0 means the <u>worst</u> health you can imagine. Please indicate on the scale how your health is TODAY.

YOUR HEALTH

TODAY =

ZARIT BURDEN INTERVIEWINSTRUCTIONS: The following is a list of statements, which reflect how people sometimes feel when taking care of another person. After each statement, indicate how often you feel that way: never, rarely, sometimes, quite frequently, or nearly always. There are no right or wrong answers.

AJ1. **1. Do you feel that because of the time you spend with your relative you don’t have enough time for yourself?**

0. Never ☐
1. Rarely ☐
2. Sometimes ☐
3. Quite Frequently ☐
4. Nearly Always ☐

AK1. **2. Do you feel stressed between caring for your relative and trying to meet other responsibilities for your family or work?**

0. Never ☐
1. Rarely ☐
2. Sometimes ☐
3. Quite Frequently ☐
4. Nearly Always ☐

AL1. **3. Do you feel angry towards your relative when you are around him/her?**

0. Never ☐
1. Rarely ☐
2. Sometimes ☐
3. Quite Frequently ☐
4. Nearly Always ☐

AM1. **4. Do you feel that your relative currently affects your relationship with other family members or friends in a negative way?**

0. Never ☐

1. Rarely ☐

2. Sometimes ☐

3. Quite Frequently ☐

4. Nearly Always ☐

AN1. **5. Do you feel strained when you are around your relative?**

0. Never ☐

1. Rarely ☐

2. Sometimes ☐

3. Quite Frequently ☐

4. Nearly Always ☐

AO1. **6. Do you feel your health has suffered because of your involvement with your relative?**

0. Never ☐

1. Rarely ☐

2. Sometimes ☐

3. Quite Frequently ☐

4. Nearly Always ☐

AP1. **7. Do you feel that you don’t have as much privacy as you would like because of your relative?**

0. Never ☐

1. Rarely ☐

2. Sometimes ☐

3. Quite Frequently ☐

4. Nearly Always ☐

AQ1. **8. Do you feel that your social life has suffered because you are caring for your relative?**

- 0. Never ☐
- 1. Rarely ☐
- 2. Sometimes ☐
- 3. Quite Frequently ☐
- 4. Nearly Always ☐

AR1. **9. Do you feel you have lost control of your life since your relative's illness?**

- 0. Never ☐
- 1. Rarely ☐
- 2. Sometimes ☐
- 3. Quite Frequently ☐
- 4. Nearly Always ☐

AS1. **10. Do you feel uncertain about what to do about your relative?**

- 0. Never ☐
- 1. Rarely ☐
- 2. Sometimes ☐
- 3. Quite Frequently ☐
- 4. Nearly Always ☐

AT1. **11. Do you feel you should be doing more for your relative?**

- 0. Never ☐
- 1. Rarely ☐

2. Sometimes ☐

3. Quite Frequently ☐

4. Nearly Always ☐

AU1. 12. Do you feel you could do a better job in caring for your relative?

0. Never ☐

1. Rarely ☐

2. Sometimes ☐

3. Quite Frequently ☐

4. Nearly Always ☐

For any information on the use of the ZBI, please contact Mapi Research Trust, Lyon, France.

Internet: https://eprovide.mapi-trust.org

AW1. Thank you for taking the time to complete this survey, we truly value the information you have provided. Your responses will contribute to our analyses and help us getting a better understanding of how Arginase 1 Deficiency impacts the healthcare resource use and the quality of life for you and your family. Before completing the survey, we would like to give you the opportunity to share additional thoughts around how Arginase 1 Deficiency impacts your and your family's life. Is there anything you think we should know?

You have now reached the end of the survey, please click submit to save your answers.

AX1. if((q2.NAOK == 1), "https://svara.enkatfabriken.com/b/index.php?r =survey/index&sid=739916&newtest=Y&lang=en&crid=", "")

The questionnaire was successfully submitted. Thank you for taking the time to complete this survey!

{if((q2.NAOK==1),"You have stated that you have more than one child with Arginase 1 Deficiency. We would now like to answer the questionnaire on behalf of your other child/children with Arginase 1 Deficiency. Please click the link below to reach the survey: ","")}
