# Supplementary material for: Disease burden among patients with Arginase 1 deficiency and their caregivers: A multinational, cross‐sectional survey
Source: JIMD Rep. 2024 Oct 29;65(6):450–60. doi: 10.1002/jmd2.12456 (PMC11540579; doi:10.1002/jmd2.12456)
Supplement: Supplementary file 2 — Data S2. Supporting information. [file JMD2-65-450-s001.pdf]

B1. Informed Consent for Adult Participants

Before you start answering the survey, we ask you to give your formal consent to participate in the "Survey of Resource Use and Health-Related Quality of Life in People with arginase 1 deficiency".

By clicking in the box below I certify that:

I have read the content in the above Patient Information Sheet (V2.0 24-03-2023) where the study is described. I have had the opportunity to consider the information, ask questions and have had these answered satisfactorily. I consent to my responses being collected in the study. I consent to my responses being treated as described in the above Patient Information Sheet. I understand that I cannot and will not be identified in any future publication or report that is based on my data. I understand that my participation is voluntary and that I can choose not to participate without giving any reason. This will in no way affect my medical care or legal rights. I am aware that I can ask for more information from the contact persons for the study or from my physician.

I give my informed consent to take part in the study:

Yes ☐

No ☐

C1. What is your age today?

☐☐

C2. What is your biological gender?

Female ☐

Male ☐

C3. What is your current living arrangement?

Living with parent(s) ☐

Live in own home ☐

Supported living services for people with disabilities ☐

Other, please specify: ☐

Other, please specify:

[ ]

C4. **How many adults (18 years and above) do you live with?**

- 0 ☐
- 1 ☐
- 2 ☐
- 3 ☐
- 4 ☐
- 5 ☐
- More than 5 ☐

C5. **How many children (below 18 years) do you live with?**

- 0 ☐
- 1 ☐
- 2 ☐
- 3 ☐
- 4 ☐
- 5 ☐
- More than 5 ☐

C6. **What is your current main occupation?**

- Primary/secondary school ☐
- Student/University ☐
- Employed or self-employed ☐
- Sick-leave or early retirement ☐
- Unemployed or looking for work ☐
- Other, please specify: ☐

Other, please specify:

C7. What is the highest level of education you have completed?

- [ ] Did not complete primary school
- [ ] Primary school
- [ ] Secondary school
- [ ] University
- [ ] Don't know
- [ ] Other, please specify:

Other, please specify:

C8. Have you received specialised education due to your Arginase 1 Deficiency diagnosis?

- [ ] No
- [ ] Don’t know
- [ ] Yes, please specify (optional)

Yes, please specify (optional)

D1. At what age did you experience your first symptom of Arginase 1 Deficiency?

- [ ] Don’t know
- [ ] Age

Age

D2. **At what age did you notice the first problem in your movement abilities due to Arginase 1 Deficiency?**

- No problems with regards to movement ☐
- Don’t know ☐
- Age: ☐

Age:

D3. **At what age did you notice the first problems in your ability to think, read, learn, remember, reason, or pay attention?**

- No problems ☐
- Don’t know ☐
- Age: ☐

Age:

D4. **What was the first problem(s) you noticed related to your ability to think, read, learn, remember, reason, or pay attention?**

D5. **At what age were you diagnosed with Arginase 1 Deficiency?**

- Diagnosed by new-born screening ☐
- Don’t know ☐
- Age: ☐

Age:

D6. **Did you receive an incorrect diagnosis before receiving your Arginase 1 Deficiency diagnose?**

Yes ☐

No ☐

Don't know ☐

D7. **What diagnosis did you receive first?**

D8. **At what age did you receive this incorrect diagnosis?**

Don't know ☐

Age: ☐

Age:

D9. **Have you received a liver transplant?**

Yes ☐

No ☐

D10. **Do you experience spasticity (stiffness of muscle, which might interfere with movement, speech, or be associated with discomfort or pain) due to Arginase 1 Deficiency? Check all that apply**

Yes, lower limbs (legs) ☐

Yes, upper limbs (arms) ☐

No ☐

Don't know ☐

D11. **Have you experienced seizures due to Arginase 1 Deficiency? (Seizures are bursts of electrical activity in the brain that temporarily affect how it works)**

Yes ☐

No ☐

Don't know ☐

D12. How many seizures have you experienced during the last 4 weeks?

- [ ] Don't know
- [ ] Number of seizures:

Number of seizures:

D13. Are you taking any medication to help control seizures (i.e. anti-epileptic drug)?

- [ ] Yes
- [ ] No
- [ ] Don't know

D14. Have you experienced any other symptoms due to Arginase 1 Deficiency?

- [ ] Yes
- [ ] No
- [ ] Don't know

D15. Please specify your other symptoms:

D16. Do you have any other long-term (6 months or more) illness or disability?

- [ ] Yes
- [ ] No
- [ ] Prefer not to say

D17. What other illness/disability do you have? Check all that apply.

- [ ] Hypertension (high blood pressure)
- [ ] Diabetes type 2
- [ ] Cerebral palsy
- [ ] Anaemia (lower than normal number of red blood cells)
- [ ] Kidney disease
- [ ] Swallowing problems

Low body weight/Eating disorder ☐

Thyroid disease ☐

Heart problems ☐

Lung diseases ☐

Muscle and bone disorder ☐

Chronic obstructive pulmonary disease (COPD) ☐

Stroke ☐

Restless legs syndrome ☐

Chronic heart failure ☐

Gastro-oesophageal reflux (relating to increased stomach acid) ☐

Depression ☐

None of the above ☐

E1. During the last 12 months, have you visited the A&E (emergency) department at the hospital due to Arginase 1 Deficiency?

Yes ☐

No ☐

Don’t know ☐

E2. How many times have you visited the A&E (emergency) department at the hospital due to Arginase 1 Deficiency during the last 12 months?

Don’t know ☐

times ☐

times

E3. During the last 12 months, have you been hospitalised due to Arginase 1 Deficiency?

Yes ☐

No ☐

Don’t know ☐

E4. **How many times have you been hospitalised due to Arginase 1 Deficiency during the last 12 months?**

E5. **For how many days were you hospitalized?1st hospitalisation:**

days

E6. **For how many days were you hospitalized?2nd hospitalisation:**

days

E7. **For how many days were you hospitalized?3rd hospitalisation**

days

E8. **For how many days were you hospitalized?4th hospitalization**

days

E9. **For how many days were you hospitalized?5th hospitalization**

days

E10. **For how many days were you hospitalized?6th hospitalization**

days

E11. **For how many days were you hospitalized?7th hospitalization**

days

E12. **For how many days were you hospitalized?8th hospitalization**

days

E13. **For how many days were you hospitalized?9th hospitalization**

days

E14. **For how many days were you hospitalized?10th hospitalization**

days

E15. **For how many days were you hospitalized?11th hospitalization**

days

E16. **For how many days were you hospitalized?12th hospitalization**

days

E17. **During the last 12 months, have you visited any of the below health care staff due to Arginase 1 Deficiency? (Do not include visits to the A&E or hospitalization that you have responded to before, check all that apply)**

General practitioner

Neurologist

Paediatrician

Metabolic specialist ☐

Nurse ☐

Physiotherapist or rehabilitation specialist ☐

Occupational therapist ☐

Psychologist ☐

Dietitian ☐

Geneticist ☐

Speech and language therapist ☐

None of the above ☐

Other ☐

Other

E18. How many visits to each of the following have you done in the last 12 months?General practitioner

Number of visits during the last 12 months ☐☐☐☐☐☐☐☐☐☐

E19. How many visits to each of the following have you done in the last 12 months?Neurologist

Number of visits during the last 12 months ☐☐☐☐☐☐☐☐☐☐

E20. How many visits to each of the following have you done in the last 12 months?Paediatrician

Number of visits during the last 12 months ☐☐☐☐☐☐☐☐☐☐

E21. How many visits to each of the following have you done in the last 12 months?Metabolic specialist

Number of visits during the last 12 months ☐☐☐☐☐☐☐☐☐☐

E22. How many visits to each of the following have you done in the last 12 months?Nurse

Number of visits during the last 12 months ☐☐☐☐☐☐☐☐☐☐

E23. How many visits to each of the following have you done in the last 12 months?Physiotherapist or rehabilitation specialist

Number of visits during the last 12 months ☐☐☐☐☐☐☐☐☐☐

E24. How many visits to each of the following have you done in the last 12 months?Occupational therapist

Number of visits during the last 12 months

E25. How many visits to each of the following have you done in the last 12 months?Psychologist

Number of visits during the last 12 months

E26. How many visits to each of the following have you done in the last 12 months?Dietitian

Number of visits during the last 12 months

E27. How many visits to each of the following have you done in the last 12 months?Geneticist

Number of visits during the last 12 months

E28. How many visits to each of the following have you done in the last 12 months?Speech and language therapist

Number of visits during the last 12 months

E29. How many visits to each of the following have you done in the last 12 months?Other {q34_other}

Number of visits during the last 12 months

E30. During the last 12 months, have you had any of the below test due to Arginase 1 Deficiency? Check all that apply

- [ ] Blood test
- [ ] Ultrasound
- [ ] Electrocardiogram (ECG) Test to evaluate the heart
- [ ] Electroencephalogram (EEG)Test to evaluate the brain/ seizure activity
- [ ] Magnetic resonance imaging (MRI) scan
- [ ] Computed tomography (CT) scan
- [ ] Don't know
- [ ] Other

Other

E31. How many tests have you done during the last 12 months?Blood test

Number of tests during the last 12 months

E32. **How many tests have you done during the last 12 months?Ultrasound**

Number of tests during the last 12 months

E33. **How many tests have you done during the last 12 months?Electrocardiogram (ECG) Test to evaluate the heart**

Number of tests during the last 12 months

E34. **How many tests have you done during the last 12 months?Electroencephalogram (EEG)Test to evaluate the brain/ seizure activity**

Number of tests during the last 12 months

E35. **How many tests have you done during the last 12 months?Magnetic resonance imaging (MRI) scan**

Number of tests during the last 12 months

E36. **How many tests have you done during the last 12 months?Computed tomography (CT) scan**

Number of tests during the last 12 months

E37. **How many tests have you done during the last 12 months?Other; {q35_other}**

Number of tests during the last 12 months

E38. **During the last 12 months, have you been treated with injections of botulinum toxin (Botox, Xeomin or Dysport) for muscle stiffness?**

Yes

No

Don’t know

E39. **How many times have you received this treatment in the last 12 months?**

Don't know

Number of treatment:

Number of treatment:

E40. **During the last 12 months, have you received surgical treatments for muscle stiffness?**

- [ ] Yes
- [ ] No
- [ ] Don't know

E41. **How many surgical treatments have you received in the last 12 months?**

- [ ] Don’t know
- [ ] Number of treatments:

Number of treatments:

F1. **Have you been prescribed a special diet to follow for your Arginase 1 Deficiency?**

- [ ] Yes
- [ ] No
- [ ] Don’t know

F2. **In the past 7 days, did you manage to adhere to this prescribed diet for your Arginase 1 Deficiency?**

- [ ] Yes, all the meals
- [ ] Most of the time
- [ ] Sometimes
- [ ] A little of the time
- [ ] No, none of the meals
- [ ] Don’t know

F3. **How easy/difficult do you think it is to follow the dietician’s and/or physician’s guidelines for your ARG1-D diet?**

- [ ] Very easy
- [ ] Easy
- [ ] Difficult
- [ ] Very difficult
- [ ] Don't know

F4. **Do you have problems with swallowing and/or self-feed? Check all that apply.**

- [ ] No, can self-feed with no problems
- [ ] Yes, problems swallowing
- [ ] Yes, problems self-feed
- [ ] Don't know

F5. **Do you regularly consume protein substitute? By protein substitute we mean an amino acid mixture prescribed by your physician/dietician.**

- [ ] Yes
- [ ] No
- [ ] Don't know

F6. **During the last 12 months, have you used any of the medicines listed below for your Arginase 1 Deficiency? Check all that apply.**

- [ ] Sodium benzoate
- [ ] Sodium phenylbutyrate (Ammonaps or Pheburane)
- [ ] Glycerol phenylbutyrate (Ravicti)
- [ ] Baclofen (muscle relaxant medication for spasticity)
- [ ]
- [ ] None of the above
- [ ] Other, please specify:

Other, please specify:

F7.

G1. **During the last 12 months, have you received professional assistance as a consequence of Arginase 1 Deficiency? Do not include assistance/caregiving by relatives or friends.**

- Yes ☐
- No ☐
- Don't know ☐

G2. **What type of professional assistance have you received? Check all that apply.**

- Daily activities (leisure, school, work) ☐
- Personal care ☐
- Household ☐
- Transportation ☐
- Other, please specify: ☐

Other, please specify:

[ ]

G3. **How many hours per week do you receive professional assistance in total?**

- Don't know ☐
- Hours of assistance per week ☐

Hours of assistance per week

[ ]

G4. **At what age did you start receiving assistance?**

- Dont know ☐
- Age ☐

Age

[ ]

G5. **Have you made any adjustments in your home as a consequence of your Arginase 1 Deficiency?**

Yes ☐

No ☐

Don't know ☐

G6. **What type of adjustments have you made?**

G7. **Do you use any mobility aids or devices due to Arginase 1 Deficiency?**

Yes ☐

No ☐

Don’t know ☐

G8. **What type of mobility aids do you use? Check all that apply.**

Wheelchair ☐

Walking aids (walker, rollator, crutches, canes) ☐

Walking stabilators (casts, splints etc.) ☐

Other, please specify: ☐

Other, please specify:

G9. **At what age did you start using the mobility aid or devices?Wheelchair**

Age when started using

G10. **At what age did you start using the mobility aid or devices?Walking aids (walker, rollator, crutches, canes)**

Age when started using

G11. **At what age did you start using the mobility aid or devices?Walking stabilators (casts, splints etc.)**

Age when started using

G12. **At what age did you start using the mobility aid or devices?Other {q47b_other}**

Age when started using ☐☐☐☐☐☐☐☐☐☐

H1. **Are you employed or self-employed?**

- Yes ☐
- No ☐
- Don’t know ☐

H2. **Are you fully employed?**

- Yes ☐
- Don’t know ☐
- No, specify the % that you work: ☐

No, specify the % that you work:

H3. **Are you on long-term sick-leave or on early retirement due to Arginase 1 Deficiency? Check all that apply.**

- Yes – sick-leave full-time ☐

Comment

- Yes – sick-leave part-time– please specify percentage ☐

Comment

- Yes - early retirement full-time ☐

Comment

- Yes – early retirement part-time – please specify percentage ☐

Comment

No

Comment

Don't know

Comment

H4. At what age did you go on sick-leave/early retirement?

H5. Have you been absent from work due to symptoms from Arginase 1 Deficiency during the last 4 weeks?

Yes

No

Don't know

H6. How many hours have you been absent from work due to symptoms from Arginase 1 Deficiency during the last 4 weeks?

Don't know

In total hours, around

In total hours, around

H7. During the past seven days, how much did your health problems affect your productivity while you were working?

Think about days you were limited in the amount or kind of work you could do, days you accomplished less than you would like, or days you could not do your work as carefully as usual. If health problems affected your work only a little, choose a low number. Choose a high number if health problems affected your work a great deal.

Consider only how much health problems affected productivity while you were working.

0 – health problems had no effect on my work	1	2	3	4	5	6	7	8	9	10 – health problems completely prevented me from working
☐	☐	☐	☐	☐	☐	☐	☐	☐	☐	☐

H8. During the past seven days, how much did your health problems affect your ability to do regular daily activities?

By regular activities, we mean the usual activities you do, such as shopping, childcare, exercising, studying, etc. Think about times you were limited in the amount or kind of activities you could do and times you accomplished less than you would like. If health problems affected your activities only a little, choose a low number. Choose a high number if health problems affected your activities a great deal.

Consider only how much health problems affected your ability to do your regular daily activities, other than work at a job.

0 – health problems had no effect on my daily activities	1	2	3	4	5	6	7	8	9	10 – health problems prevented me from doing my daily activities
☐	☐	☐	☐	☐	☐	☐	☐	☐	☐	☐

I1. MOBILITY

- I have no problems in walking about ☐
- I have slight problems in walking about ☐
- I have moderate problems in walking about ☐
- I have severe problems in walking about ☐
- I am unable to walk about ☐

J1. SELF-CARE

- I have no problems washing or dressing myself ☐
- I have slight problems washing or dressing myself ☐
- I have moderate problems washing or dressing myself ☐
- I have severe problems washing or dressing myself ☐
- I am unable to wash or dress myself ☐

K1. USUAL ACTIVITIES (e.g. work, study, housework, family or leisure activities)

- I have no problems doing my usual activities ☐
- I have slight problems doing my usual activities ☐
- I have moderate problems doing my usual activities ☐
- I have severe problems doing my usual activities ☐
- I am unable to do my usual activities ☐

L1. PAIN/ DISCOMFORT

I have no pain or discomfort ☐

I have slight pain or discomfort ☐

I have moderate pain or discomfort ☐

I have severe pain or discomfort ☐

I have extreme pain or discomfort ☐

M1. ANXIETY / DEPRESSION

I am not anxious or depressed ☐

I am slightly anxious or depressed ☐

I am moderately anxious or depressed ☐

I am severely anxious or depressed ☐

I am extremely anxious or depressed ☐

N1. We would like to know how good or bad your health is TODAY. This scale is numbered from 0 to 100. 100 means the best health you can imagine.
0 means the worst health you can imagine. Please indicate on the scale how your health is TODAY.

YOUR HEALTH

TODAY =

O1. Thank you for taking the time to complete this survey, we truly value the information you have provided. Your responses will contribute to our analyses and help us getting a better understanding of how Arginase 1 Deficiency impacts the healthcare resource use and the quality of life for you and your family.

Before completing the survey, we would like to give you the opportunity to share additional thoughts around how Arginase 1 Deficiency impacts your and your family's life. Is there anything you think we should know?

Thank you for taking the time to respond to this questionnaire! If you have a caregiver, he/she is welcome to answer a similar questionnaire by clicking here: CAREGIVER SURVEY

If your caregiver wants to respond to the questionnaire later, please follow the instructions below: Open the link to the CAREGIVER SURVEY Click on the "Resume later" button in the top right corner Add username and password for the survey Your caregiver can respond to the questionnaire later by opening the link to the questionnaire, click on the "Load unfinished survey" button at the first page and then enter the username and password.
